# Editorial: Applications of AI and machine learning in finance and economics

**DOI:** 10.3389/frai.2025.1715929

**Published:** 2025-10-21

**Authors:** Alessia Paccagnini, Maria Iannario, Joerg Osterrieder, Adamaria Perrotta, Fabio Parla, Hanna Kristín Skaftadóttir

**Affiliations:** ^1^University College Dublin, Dublin, Ireland; ^2^Università degli Studi di Napoli Federico II, Naples, Italy; ^3^Universiteit Twente, Enschede, Netherlands; ^4^Università degli Studi di Palermo, Palermo, Italy; ^5^Haskolinn a Bifrost, Borgarnes, Iceland

**Keywords:** FinTech, diversity and inclusion, artificial intelligence, big data and analytics, forecasting, economics

## Introduction

The rapid evolution of AI and Machine Learning is driving a major transformation in finance and economics, enabling real-time data processing, improved decision-making, and advanced risk management. This Research Topic, developed in partnership with the Women in FinTech and AI 2024 conference and MSCA Industrial Doctoral Network on Digital Finance (Grant agreement No: 101119635), highlights cutting-edge research reshaping how we understand and operate in financial markets. The articles included in this Research Topic shed some light in a number of areas where finance, advanced statistical models, and economics are facilitating AI and ML to make significant contributions.

Big Data Analytics in Finance: The enormous increase in data production has opened up huge potential in financial analysis. Today, complex patterns in the huge volumes of data can now be processed and interpreted by advanced ML algorithms to allow us to gain insights about the market sentiment, economic indicators, systemic risks, as well as other factors that a traditional analysis cannot pick up. This Research Topic illustrates this point through compelling contributions such as “*Predicting the Bitcoin's price using AI*” (Cohen and Aiche), which explores the application of AI and ML in predicting Bitcoin price movements and design adaptive strategies. The study highlights how these technologies are becoming essential for ensuring stability and uncovering opportunities in increasingly interconnected global markets.

Natural Language Processing in Economic Analysis: The use of NLP in understanding financial texts, news items, social sentiment information, and regulatory reports, is transforming the way we know the financial markets, and economic stories. This Research Topic presents compelling research in the field, including contributions such as the article on “*Does business news sentiment matter in the energy stock market? Globalization and the proliferation of sentiment analysis in extracting short-term stock market prediction*” (Lee and Anderl), which explains how sentiment analysis is found to be an efficient method of analysis in making short-term predictions in the stock market concerning the energy industry which is considered a volatile market. Also, the work on “*NLP-augmented inflation measurement with BERT and web scraping*” (Berki et al.) demonstrates how transformer-based models can effectively categorize product data and monitor inflation in close to a real time setting based on unstructured text, enhancing established metrics with novel dimensions of information.

Digital Finance and Sustainability: One particularly promising area lies at the intersection of sustainable finance and artificial intelligence. Machine learning tools are increasingly being applied to assess goods recycling, evaluate environmental, social, and governance (ESG) factors, optimize green investment portfolios, and design innovative financial products that support the advancement of the Sustainable Development Goals. The articles in this Research Topic discuss the role of this convergence in meeting the increasing need of responsible finance by using technology to measure and manage sustainability risks and opportunities. Moreover, the research also covers some specialized fields, such as “*AI revolution in insurance: bridging research and reality*” (Bhattacharya et al.), that already covers all important aspects of the field (automotive, health, and property insurance) because the segment is ready to explore the field as innovative and environmental friendly.

AI-Powered Financial Markets: Intelligent portfolio management systems, robo-advisors and algorithmic trading are transforming the way the financial markets are being played. The systems enable orders, digital information processing and trade execution in areas a human trader could never operate, at such velocity and magnitude, but also give consideration to complicated risk tolerances and regulative restrictions. In this selection, the entitled *Explainable machine learning to predict the cost of capital* (Bussmann et al.) addresses the most salient issue of these innovations, namely interpretability of AI-driven financial estimations, and the article *Predicting financial distress in TSX-listed firms using machine learning algorithms* (Lokanan and Ramzan), pertains to a more applied issue of the risk assessment. Such studies demonstrate how innovations lead to a more efficient price discovery, a better liquidity level and result in better market access but without losing transparency and regulatory compliance.

Blockchain and Distributed Ledger Applications: The combination of AI and the blockchain is opening new opportunities of conducting safe, transparent, and efficient financial transactions. Artificial intelligence-based smart contracts have the potential to automate more sophisticated financial agreements, and blockchain infrastructure offers the security measures and immutability required by high-stakes financial processes. This discussion about the institutional adoption challenges, including in the form of AI and ML in banking systems, in the form of a qualitative survey of board of directors, is proposed in: *Adoption of artificial intelligence and machine learning in banking systems: a qualitative survey of board of directors* (Eskandarany).

## The role of diversity and inclusion

The partnership of this Research Topic with the conferences Women in FinTech and AI 2024 highlights the issue of diversity and inclusion in promoting innovative changes in the financial technology market. As we continue to see the future of finance entirely revolve around AI and ML, these technological advancements must be developed and implemented by diverse groups, ensuring that we create systems that offer equal benefits to all stakeholders. The article *Segmenting female students perceptions about Fintech using Explainable AI* (Adam) (winner of the Best Paper Award of 2024 edition) suggests the use of Financial Technology (Fintech) would be another potential feature in bridging the gender gap with regards to Finance and socially as well.

## Forecasting business cycle and financial indicators

ML and AI are coming to play an important role in the future of finance and economics by providing the techniques to ensure the accuracy of forecasts, at least in the data-impoverished settings. Here, two articles present new ways of predicting GDP in The Gambia, which involves applying sophisticated AI models. The first one, *GDP prediction of The Gambia using generative adversarial networks* (Jallow, Gibba et al.), with the help of generative adversarial networks uses Generative Adversarial Networks (GANs) to make highly accurate GDP predictions. The second one, *Transfer learning for predicting of gross domestic product growth based on remittance inflows using RNN-LSTM hybrid model: a case study of The Gambia* (Jallow, Mwangi et al.) uses remittance inflows as the key variables affecting a country in terms of its economic growth, as well as a hybrid version of RNN-LSTM model with transfer learning. Together, these studies highlight the growing importance of AI-driven, data-efficient forecasting tools in economic analysis, offering valuable support for policymaking and planning in fast-developing economies.

## Conclusion

The contributions in this Research Topic underscore how AI and machine learning are not merely transforming finance and economics today, but are paving the way for a future characterized by greater financial inclusion, more efficient and transparent markets, and sustainable economic growth. Looking ahead, the continued dialogue between academia, industry, and policymakers will be vital to turning technological advances into meaningful societal progress. As the field evolves, the grand challenge will be to harness innovation responsibly—ensuring that breakthroughs in AI and FinTech are guided by ethical principles and inclusivity, so that the benefits of this new financial era are shared broadly and equitably.

